# Supplementary figures and images for: Dual transcriptome based reconstruction of Salmonella-human integrated metabolic network to screen potential drug targets
Source: PLoS One. 2022 May 24;17(5):e0268889. doi: 10.1371/journal.pone.0268889 (PMC9129043; doi:10.1371/journal.pone.0268889)

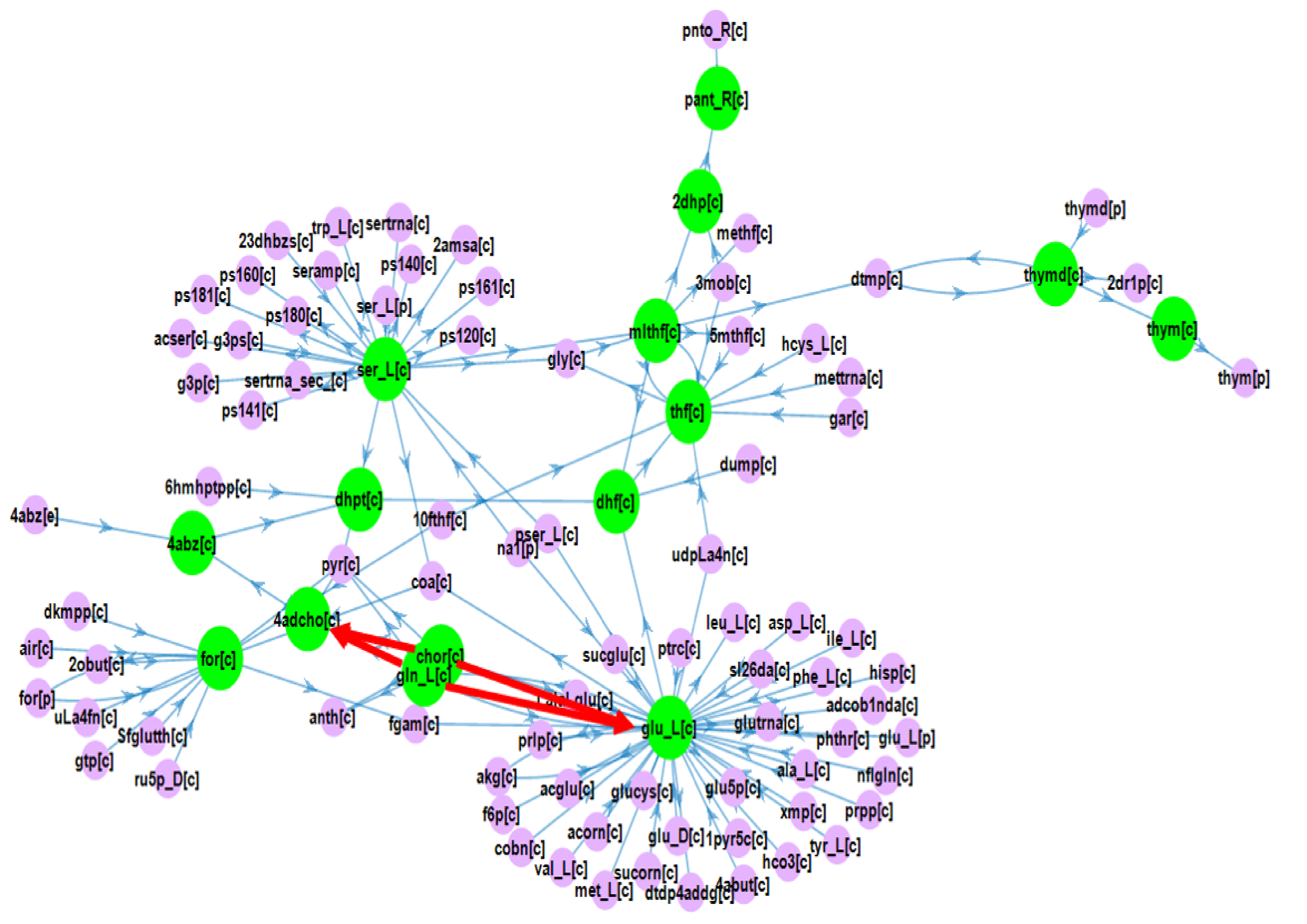

Supplement: S1 Fig — The red arrows indicate the reaction controlled by pabB (L-Glutamine + chorismate -> L-Glutamate + 4-amino-4-deoxychorismate). (TIF) [file pone.0268889.s001.tif]

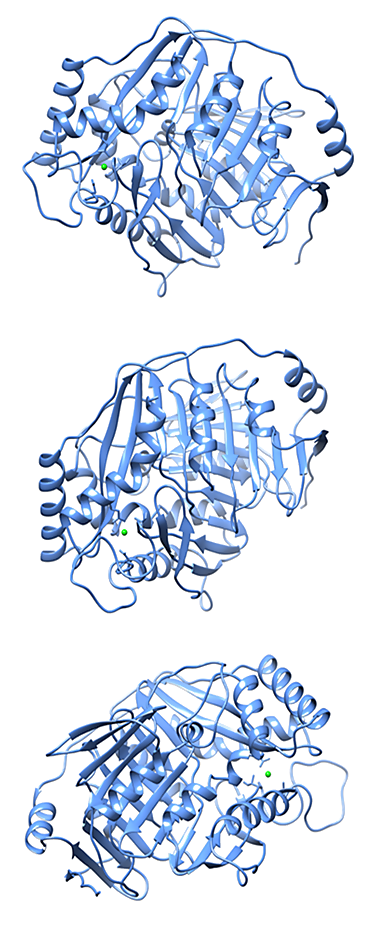

Supplement: S2 Fig — (TIF) [file pone.0268889.s002.tif]

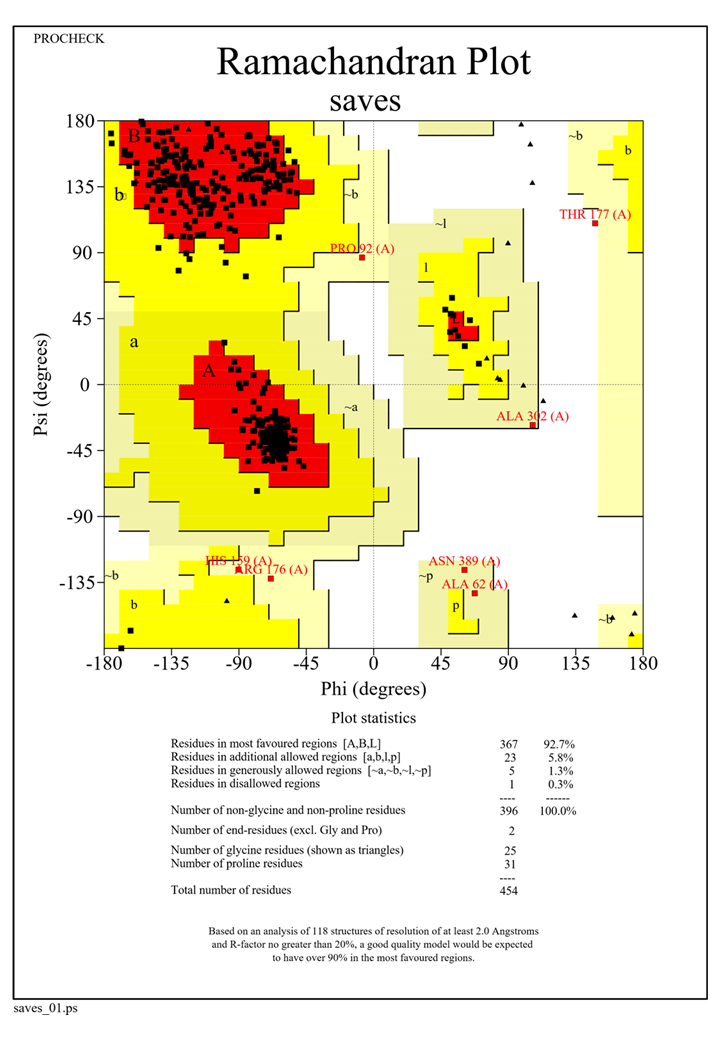

Supplement: S3 Fig — (TIF) [file pone.0268889.s003.tif]

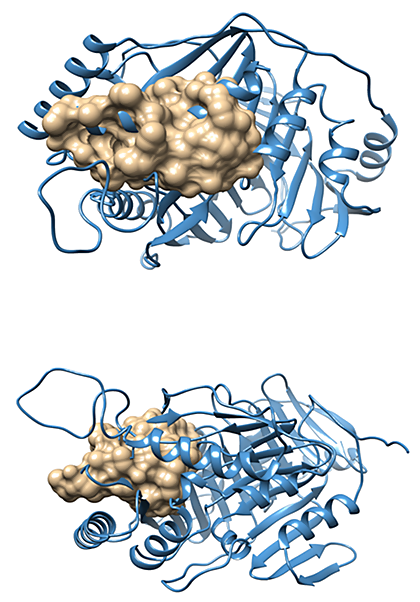

Supplement: S4 Fig — From the figure, it can be seen that Mg2+ ion is submerged within the predicted binding pocket. (TIF) [file pone.0268889.s004.tif]

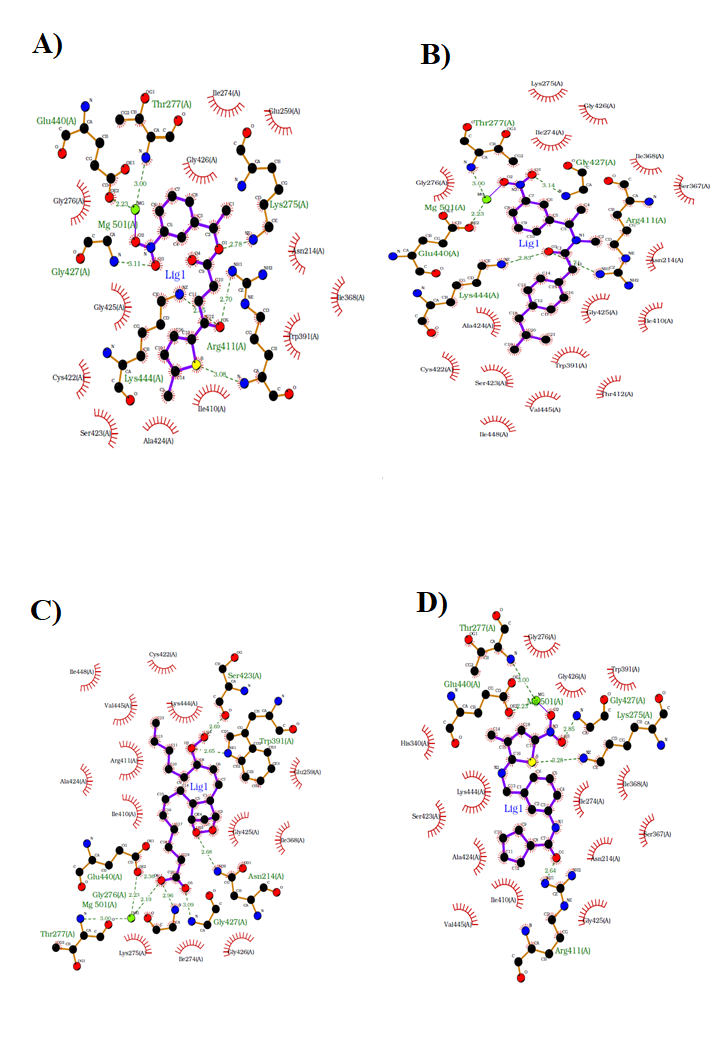

Supplement: S5 Fig — A) is representing the LigPlot+ of P12680 showing atomic interaction between protein (residues/ Mg2+ ion) and ligand (ZINC7879733). The atomic linkages due to hydrogen bonding can be identified from the diagram. Similarly, B) represents the LigPlot+ analysis of P12680 and ligand (ZINC15179659), C) represents the LigPlot+ for P12680 and ligand (ZINC14880941), and D) represents the LigPlot+ for P12680 and ligand (ZINC58542694). (TIF) [file pone.0268889.s005.tif]

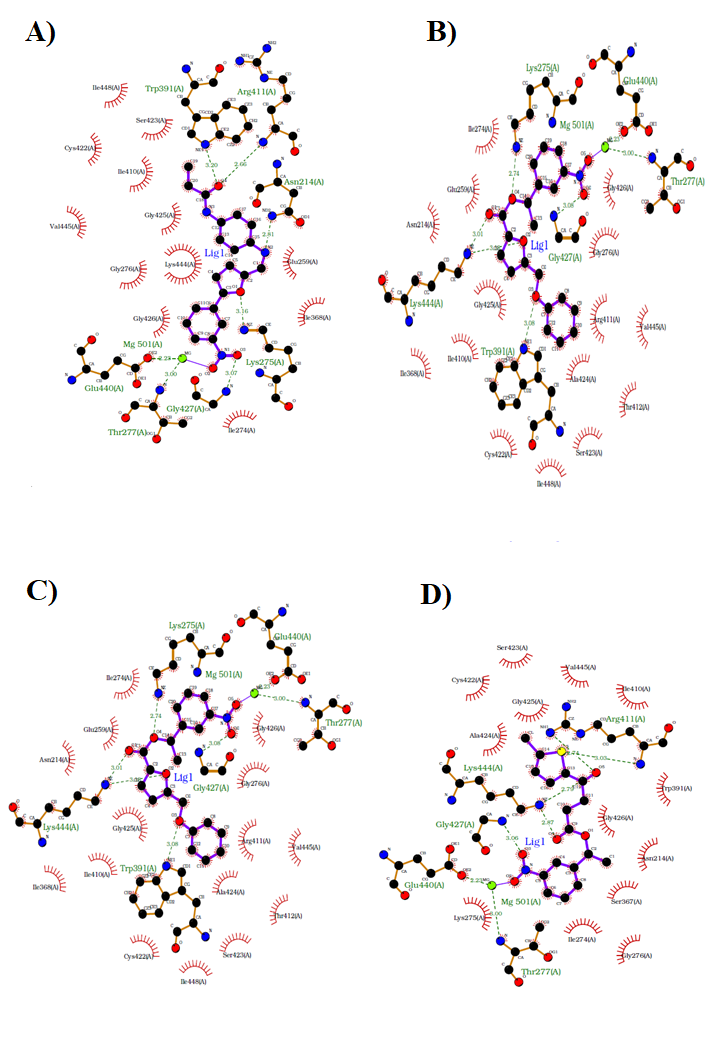

Supplement: S6 Fig — A) is the LigPlot+ for P12680 and ligand (ZINC1201089024), all the atomic linkages occurring between the protein-ligand complex can be analyzed. Likewise, B) represents the LigPlot+ for P12660 and ligand (ZINC27071723), C) is LigPlot+ for P12680 and ligand (ZINC7133393) and D) is LigPlot+ for P12680 and ligand (ZINC7879735). (TIF) [file pone.0268889.s006.tif]

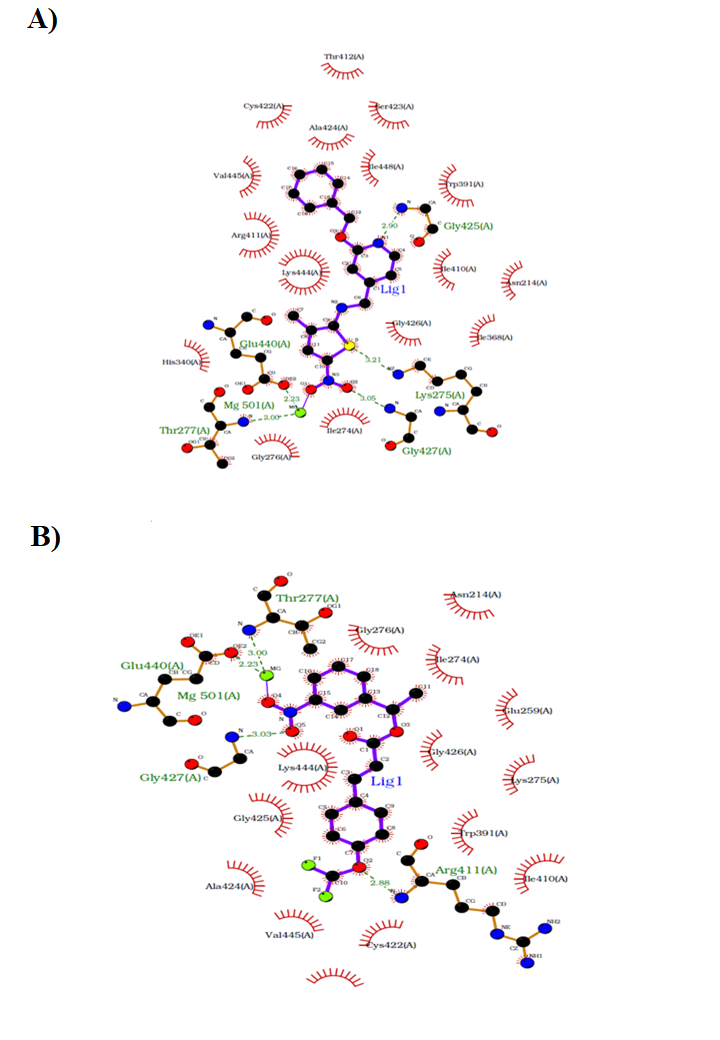

Supplement: S7 Fig — A) is the LigPlot+ for P12680 and ligand (ZINC58542238) complex and B) is the LigPlot+ for P12680 and ligand (ZINC7538530) complex. The overall amino acid residues of P12680 which interact with each of the top ten compounds (ligands) making hydrogen bonds and hydrophobic contacts. (TIF) [file pone.0268889.s007.tif]

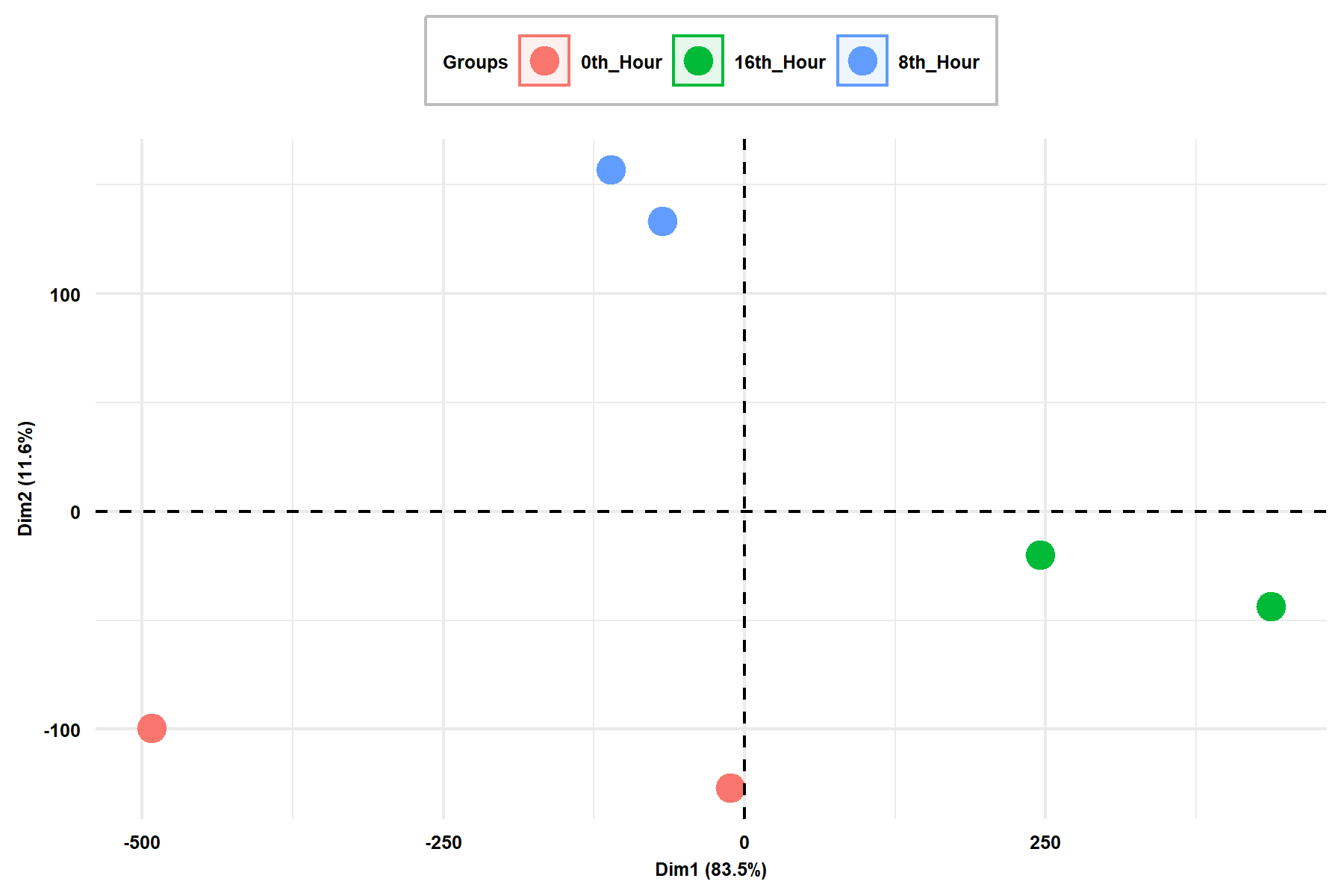

Supplement: S8 Fig — The red, blue and green dots represent beginning of infection, 8th hour of infection and 16th hour of infection respectively. (TIF) [file pone.0268889.s008.tif]

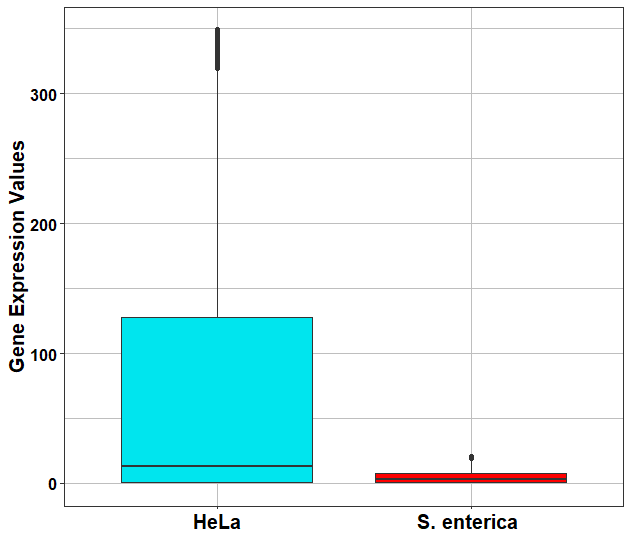

Supplement: S9 Fig — (TIFF) [file pone.0268889.s009.tiff]
