# Supplementary material for: Dual transcriptome based reconstruction of Salmonella-human integrated metabolic network to screen potential drug targets
Source: PLoS One. 2022 May 24;17(5):e0268889. doi: 10.1371/journal.pone.0268889 (PMC9129043; doi:10.1371/journal.pone.0268889)
Supplement: S1 Table — (DOCX) [file pone.0268889.s010.docx]

S1 Table. 89 potential drug targets that are not similar to human proteins.

| **Locus Names** | **Genename** | **Locus Names** | **Genename** | **Locus Names** | **Genename** |
| --- | --- | --- | --- | --- | --- |
| *STM0045* | *ribF* | *STM2082* | *rfbP* | *STM3862* | *glmU* |
| *STM0064* | *dapB* | *STM2085* | *rfbN* | *STM3904* | *ilvD* |
| *STM0087* | *folA* | *STM2086* | *rfbU* | *STM3909* | *ilvC* |
| *STM0113* | *leuA* | *STM2087* | *rfbV* | *STM3926* | *wzxE* |
| *STM0123* | *murE* | *STM2089* | *rfbJ* | *STM3947* | *dapF* |
| *STM0124* | *murF* | *STM2090* | *rfbH* | *STM4121* | *argC* |
| *STM0125* | *mraY* | *STM2091* | *rfbG* | *STM4122* | *argB* |
| *STM0126* | *murD* | *STM2093* | *rfbI* | *STM4131* | *murI* |
| *STM0128* | *murG* | *STM2094* | *rmlC* | *STM4137* | *murB* |
| *STM0129* | *murC* | *STM2232* | *oafA* | *STM4139* | *coaA* |
| *STM0134* | *lpxC* | *STM2366* | *accD* | *STM4459* | *pyrI* |
| *STM0165* | *speD* | *STM2384* | *aroC* | *STM3718* | *rfaI* |
| *STM0171* | *yadF* | *STM2652* | *pssA* | *STM3719* | *rfaB* |
| *STM0183* | *folK* | *STM2669* | *tyrA* | *STM3720* | *yibR* |
| *STM0207* | *mtn* | *STM2992* | *argA* | *STM3721* | *rfaP* |
| *STM0213* | *dapD* | *STM3195* | *ribB* | *STM3722* | *rfaG* |
| *STM0226* | *lpxD* | *STM3206* | *folB* | *STM3723* | *rfaQ* |
| *STM0228* | *lpxA* | *STM3295* | *folP* | *STM3724* | *kdtA* |
| *STM0229* | *lpxB* | *STM3307* | *murA* | *STM3725* | *coaD* |
| *STM0248* | *yaeD* | *STM3316* | *yrbI* | *STM3733* | *pyrE* |
| *STM0310* | *gmhA* | *STM3486* | *aroB* | *STM1889* | *msbB* |
| *STM0416* | *ribD* | *STM3539* | *asd* | *STM3715* | *rfaZ* |
| *STM0417* | *ribH* | *STM3692* | *lctP* | *STM3716* | *rfaY* |
| *STM0535* | *lpxH* | *STM3710* | *rfaD* | *STM1710* | *pgpB* |
| *STM0978* | *aroA* | *STM3711* | *rfaF* | *STM1711* | *ribA* |
| *STM0985* | *lpxK* | *STM3712* | *rfaC* | *STM1772* | *kdsA* |
| *STM0988* | *kdsB* | *STM3713* | *rfaL* | *STM1824* | *pabB* |
| *STM1155* | *htrB* | *STM3714* | *rfaK* |  |  |
| *STM1198* | *pabC* | *STM1358* | *aroD* |  |  |
| *STM1200* | *tmk* | *STM1426* | *ribE* |  |  |
| *STM1332* | *rfc* | *STM1707* | *pyrF* |  |  |
