# Supplementary material for: Dual transcriptome based reconstruction of Salmonella-human integrated metabolic network to screen potential drug targets
Source: PLoS One. 2022 May 24;17(5):e0268889. doi: 10.1371/journal.pone.0268889 (PMC9129043; doi:10.1371/journal.pone.0268889)
Supplement: S3 Table — (DOCX) [file pone.0268889.s012.docx]

S3 Table. Potential drug targets that have high affinity to bind drug-like molecules

| **Locus Names** | **Gene names** | **Locus Names** | **Gene names** | **Locus Names** | **Gene names** |
| --- | --- | --- | --- | --- | --- |
| *STM0045* | *ribF* | *STM1772* | *kdsA* | *STM3725* | *coaD* |
| *STM0064* | *dapB* | *STM1824* | *pabB* | *STM3733* | *pyrE* |
| *STM0087* | *folA* | *STM2090* | *rfbH* | *STM3862* | *glmU* |
| *STM0123* | *murE* | *STM2091* | *rfbG* | *STM4122* | *argB* |
| *STM0124* | *murF* | *STM2093* | *rfbI* | *STM4131* | *murI* |
| *STM0126* | *murD* | *STM2094* | *rmlC* | *STM4137* | *murB* |
| *STM0128* | *murG* | *STM2384* | *aroC* | *STM4139* | *coaA* |
| *STM0129* | *murC* | *STM2992* | *argA* | *STM1200* | *tmk* |
| *STM0134* | *lpxC* | *STM3295* | *folP* | *STM1358* | *aroD* |
| *STM0183* | *folK* | *STM3307* | *murA* | *STM1426* | *ribE* |
| *STM0207* | *mtn* | *STM3316* | *yrbI* | *STM1707* | *pyrF* |
| *STM0213* | *dapD* | *STM3486* | *aroB* | *STM1198* | *pabC* |
| *STM0228* | *lpxA* | *STM3539* | *asd* | *STM0978* | *aroA* |
| *STM0310* | *gmhA* | *STM3710* | *rfaD* |  |  |
| *STM0417* | *ribH* | *STM0988* | *kdsB* |  |  |
