# Supplementary material for: Dual transcriptome based reconstruction of Salmonella-human integrated metabolic network to screen potential drug targets
Source: PLoS One. 2022 May 24;17(5):e0268889. doi: 10.1371/journal.pone.0268889 (PMC9129043; doi:10.1371/journal.pone.0268889)
Supplement: S4 Table — The last column indicates broad spectrum analysis results. (DOCX) [file pone.0268889.s013.docx]

S4 Table. The list of final potential drug targets. The last column indicates broad spectrum analysis results.

| **Locus Names** | **Gene names** | **Protein Name** | **Pathway** | **Number of species** |
| --- | --- | --- | --- | --- |
| *STM1824* | *pabB* | Aminodeoxychorismate synthase component 1 | Folate biosynthesis | 61 |
| *STM0087* | *folA* | Dihydrofolate reductase | Folate biosynthesis | 50 |
| *STM0183* | *folK* | 2-amino-4-hydroxy-6-hydroxymethyldihydropteridine pyrophosphokinase | Folate biosynthesis | 50 |
| *STM3295* | *folP* | Dihydropteroate synthase | Folate biosynthesis | 77 |
| *STM0064* | *dapB* | 4-hydroxy-tetrahydrodipicolinate reductase | Biosynthesis of amino acids.Lysine biosynthesis | 51 |
| *STM0213* | *dapD* | 2,3,4,5-tetrahydropyridine-2,6-dicarboxylate N-succinyltransferase | Biosynthesis of amino acids.Lysine biosynthesis | 41 |
| *STM0207* | *mtnN* | 5'-methylthioadenosine/S-adenosylhomocysteine nucleosidase | Biosynthesis of amino acids. Cysteine and methionine metabolism. | 45 |
| *STM3486* | *aroB* | 3-dehydroquinate synthase | Biosynthesis of amino acids. Phenylalanine, tyrosine and tryptophan biosynthesis | 79 |
| *STM2384* | *aroC* | Chorismate synthase | Biosynthesis of amino acids. Phenylalanine, tyrosine and tryptophan biosynthesis | 75 |
| *STM3862* | *glmU* | Bifunctional protein GlmU | Amino sugar and nucleotide sugar metabolism | 82 |
| *STM2094* | *rmlC* | dTDP-4-dehydrorhamnose 3,5-epimerase | Polyketide sugar unit biosynthesis.Streptomycin biosynthesis | 50 |
| *STM1772* | *kdsA* | 2-dehydro-3-deoxyphosphooctonate aldolase | Lipopolysaccharide biosynthesis | 59 |
| *STM3316* | *kdsC* | 3-deoxy-D-manno-octulosonate 8-phosphate phosphatase KdsC | Lipopolysaccharide biosynthesis | 43 |
| *STM0988* | *kdsB* | 3-deoxy-manno-octulosonate cytidylyltransferase | Lipopolysaccharide biosynthesis | 53 |
| *STM0310* | *gmhA* | Phosphoheptose isomerase | Lipopolysaccharide biosynthesis | 45 |
| *STM0228* | *lpxA* | Acyl-[acyl-carrier-protein]--UDP-N-acetylglucosamine O-acyltransferase | Lipopolysaccharide biosynthesis | 59 |
| *STM0134* | *LpxC* | UDP-3-O-acyl-N-acetylglucosamine deacetylase | Lipopolysaccharide biosynthesis | 57 |
| *STM1200* | *tmk* | Thymidylate kinase | Pyrimidine metabolism | 53 |
| *STM1707* | *pyrF* | Orotidine 5'-phosphate decarboxylase | Pyrimidine metabolism | 49 |
| *STM1426* | *ribE* | Riboflavin synthase, alpha chain | Riboflavin metabolism | 64 |
| *STM0417* | *ribH* | 6,7-dimethyl-8-ribityllumazine synthase | Riboflavin metabolism | 68 |
| *STM0045* | *ribF* | Riboflavin biosynthesis protein | Riboflavin metabolism | 56 |
| *STM3307* | *murA* | UDP-N-acetylglucosamine 1-carboxyvinyl transferase | Peptidoglycan biosynthesis. Amino sugar and nucleotide sugar metabolism | 84 |
| *STM0129* | *murC* | UDP-N-acetylmuramate--L-alanine ligase | Peptidoglycan biosynthesis. D-Glutamine and D-glutamate metabolism | 59 |
| *STM0123* | *murE* | UDP-N-acetylmuramoyl-L-alanyl-D-glutamate--2,6-diaminopimelate ligase | Peptidoglycan biosynthesis.Lysine biosynthesis | 63 |
| *STM0128* | *murG* | UDP-N-acetylglucosamine--N-acetylmuramyl-(pentapeptide) pyrophosphoryl-undecaprenol N-acetylglucosamine transferase | Peptidoglycan biosynthesis | 47 |
| *STM0124* | *murF* | UDP-N-acetylmuramoyl-tripeptide--D-alanyl-D-alanine ligase | Peptidoglycan biosynthesis.Lysine biosynthesis | 40 |
| *STM3725* | *coaD* | Phosphopantetheine adenylyltransferase | Pantothenate and CoA biosynthesis | 82 |
