# Supplementary material for: Dual transcriptome based reconstruction of Salmonella-human integrated metabolic network to screen potential drug targets
Source: PLoS One. 2022 May 24;17(5):e0268889. doi: 10.1371/journal.pone.0268889 (PMC9129043; doi:10.1371/journal.pone.0268889)
Supplement: S5 Table — (DOCX) [file pone.0268889.s014.docx]

**S5 Table.** Table comprise of the Binding Energy, Zinc IDs, 1D and 2D structure of each of top 10 compounds.

| **Compound** | **Binding Free Energy** | **ZINC ID** | **STRINGS** | **Structure** |
| --- | --- | --- | --- | --- |
| fj25960 | -12.42 | ZINC7879733 | O([C@@H](C)c1cc(ccc1)N(=O)=O)C(=O)CCC(=O)c1sc(Cl)cc1 | 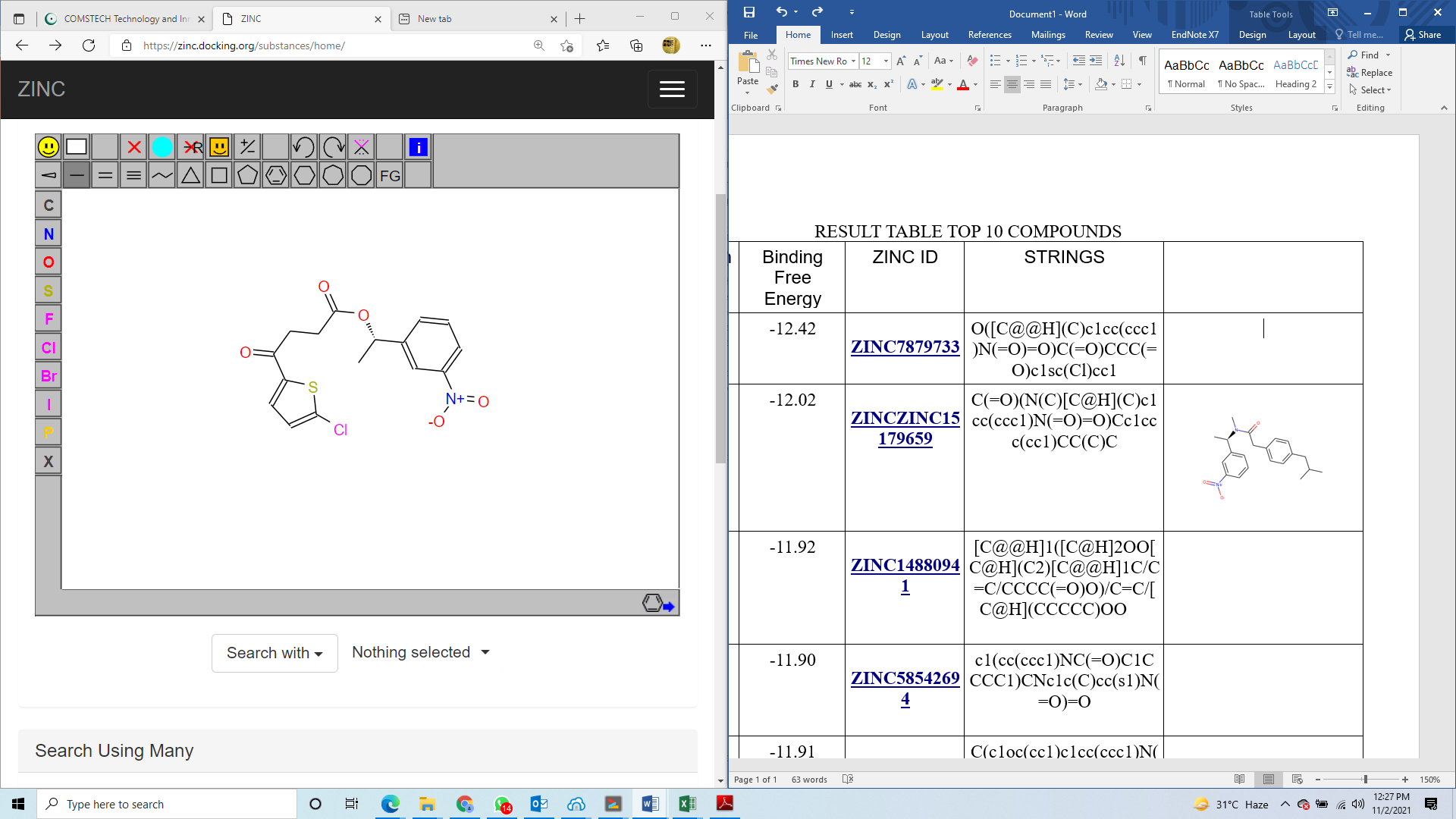 |
| fj43323 | -12.02 | ZINC15179659 | C(=O)(N(C)[C@H](C)c1cc(ccc1)N(=O)=O)Cc1ccc(cc1)CC(C)C | 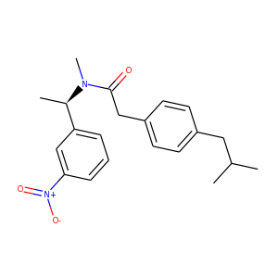 |
| fj42569 | -11.92 | ZINC14880941 | [C@@H]1([C@H]2OO[C@H](C2)[C@@H]1C/C=C/CCCC(=O)O)/C=C/[C@H](CCCCC)OO | 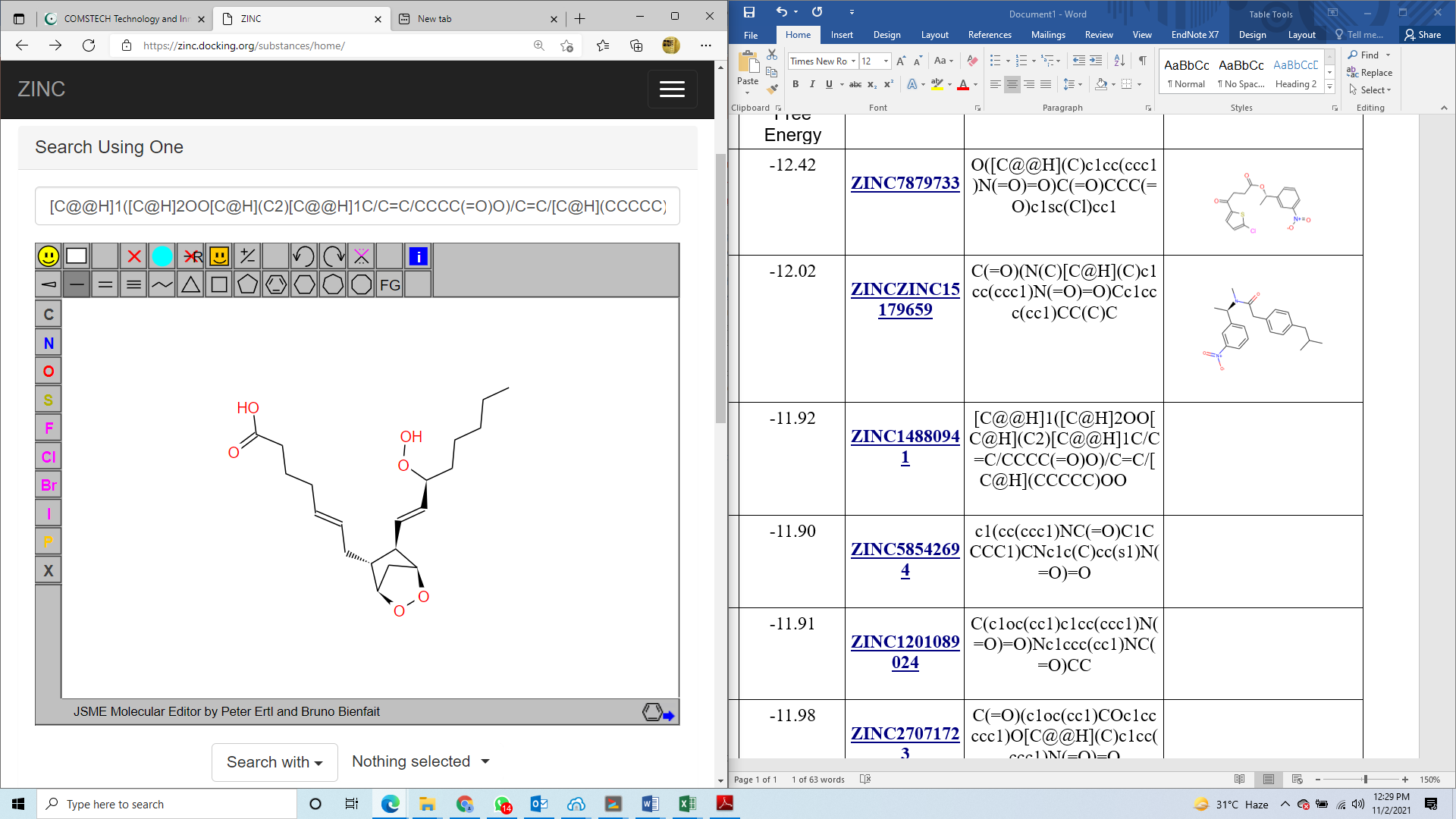 |
| fj148389 | -11.90 | ZINC58542694 | c1(cc(ccc1)NC(=O)C1CCCC1)CNc1c(C)cc(s1)N(=O)=O | 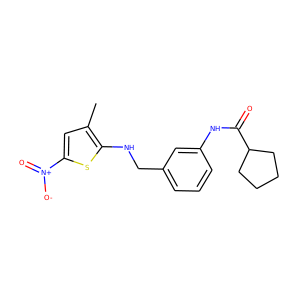 |
| fj152545 | -11.91 | ZINC1201089024 | C(c1oc(cc1)c1cc(ccc1)N(=O)=O)Nc1ccc(cc1)NC(=O)CC | 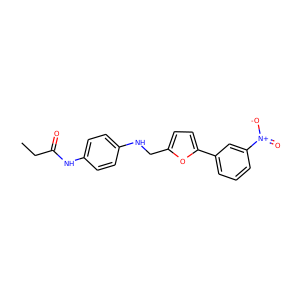 |
| fj67060 | -11.98 | ZINC27071723 | C(=O)(c1oc(cc1)COc1ccccc1)O[C@@H](C)c1cc(ccc1)N(=O)=O | 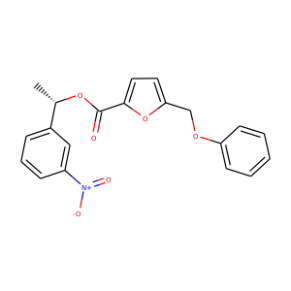 |
| fj22148 | -11.77 | ZINC7133393 | C(C(=O)Nc1cc2CCCc2cc1)S[C@@H](C)c1cc(ccc1)N(=O)=O | 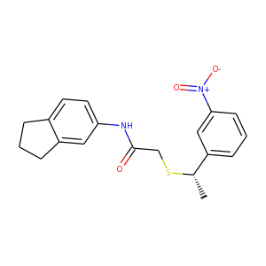 |
| fj25961 | -11.73 | ZINC7879735 | O([C@H](C)c1cc(ccc1)N(=O)=O)C(=O)CCC(=O)c1sc(Cl)cc1 | 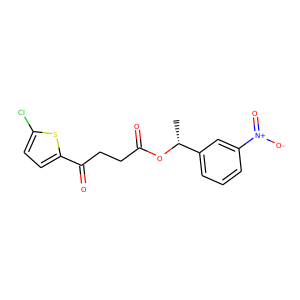 |
| fj148379 | -11.42 | ZINC58542238 | c1(cc(ncc1)OCc1ccccc1)CNc1c(C)cc(s1)N(=O)=O | 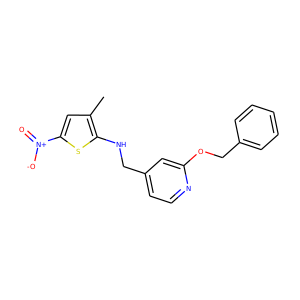 |
| fj24193 | -11.72 | ZINC7538530 | C(=O)(/C=C/c1ccc(cc1)OC(F)F)O[C@@H](C)c1cc(ccc1)N(=O)=O | 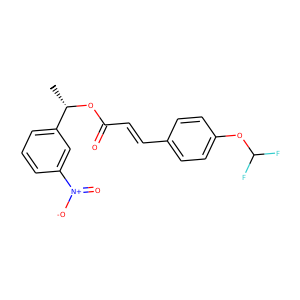 |
