# Supplementary material for: Dual transcriptome based reconstruction of Salmonella-human integrated metabolic network to screen potential drug targets
Source: PLoS One. 2022 May 24;17(5):e0268889. doi: 10.1371/journal.pone.0268889 (PMC9129043; doi:10.1371/journal.pone.0268889)
Supplement: S6 Table — (DOCX) [file pone.0268889.s015.docx]

S6 Table. Metabolites that can be consumed by *S*. Typhimurium inside host cytoplasm in pathogen-host GMN model.

| MetNames | Mets | Lower bound | Upper bound |
| --- | --- | --- | --- |
| N_Acetyl_D_glucosamine | acgam[e] | -1 | 1000 |
| Adenosine | adn[e] | -1 | 1000 |
| L_Alanine | ala_L[e] | -1 | 1000 |
| L_Arginine | arg_L[e] | -1 | 1000 |
| L_Asparagine | asn_L[e] | -1 | 1000 |
| L_Aspartate | asp_L[e] | -1 | 1000 |
| CO2 | co2[e] | -1000 | 1000 |
| L_Cysteine | cys_L[e] | -1 | 1000 |
| Decanoate_n_C100 | dca[e] | -1 | 1000 |
| Dodecanoate_n_C120 | ddca[e] | -1 | 1000 |
| Glucose | glc_D[e] | -5 | 1000 |
| Gluconate | glcn[e] | -1 | 1000 |
| L_Glutamine | gln_L[e] | -1 | 1000 |
| L_Glutamate | glu_L[e] | -1 | 1000 |
| Glycerol | glyc[e] | -1 | 1000 |
| H | h[e] | -5 | 1000 |
| H2O | h2o[e] | -1000 | 1000 |
| Hexadecanoate_n_C160 | hdca[e] | -1 | 1000 |
| Hexadecenoate_n_C161 | hdcea[e] | -1 | 1000 |
| L_Histidine | his_L[e] | -1 | 1000 |
| L_Isoleucine | ile_L[e] | -1 | 1000 |
| L_Lysine | lys_L[e] | -1 | 1000 |
| L_Methionine | met_L[e] | -1 | 1000 |
| Nicotinic acid | nac[e] | -1 | 1000 |
| Nicotinamide mononucleotide | nmn[e] | -1 | 1000 |
| O2 | o2[e] | -1 | 1000 |
| octadecanoate_n_C180 | ocdca[e] | -1 | 1000 |
| Octadecenoate (n_C18:1) | ocdcea[e] | -1 | 1000 |
| L_Phenylalanine | phe_L[e] | -1 | 1000 |
| Phosphate | pi[e] | -5 | 1000 |
| Pantothenate | pnto_R[e] | -1 | 1000 |
| L_Proline | pro_L[e] | -1 | 1000 |
| Pyridoxal | pydx[e] | -1 | 1000 |
| L_Serine | ser_L[e] | -1 | 1000 |
| Thiamin | thm[e] | -1 | 1000 |
| L_Threonine | thr_L[e] | -1 | 1000 |
| L_Tryptophane | trp_L[e] | -1 | 1000 |
| tetradecanoate_n_C140 | ttdca[e] | -1 | 1000 |
