# Supplementary material for: Dual transcriptome based reconstruction of Salmonella-human integrated metabolic network to screen potential drug targets
Source: PLoS One. 2022 May 24;17(5):e0268889. doi: 10.1371/journal.pone.0268889 (PMC9129043; doi:10.1371/journal.pone.0268889)
Supplement: S7 Table — (DOCX) [file pone.0268889.s016.docx]

S7 Table. The exchange metabolites of *S*. Typhimurium that can be found inside cytoplasm of human cell.

| **S. Typhimurium met names** | **ihsa met names** | **S. Typhimurium met names** | **ihsa met names** | **S. Typhimurium met names** | **ihsa met names** |
| --- | --- | --- | --- | --- | --- |
| 23cump[e] | m00570[c] | crn[e] | m02348[c] | glc_D[e] | m01965[c] |
| 2pg[e] | m00674[c] | csn[e] | m01632[c] | glcn[e] | m01683[c] |
| 34dhpac[e] | m00728[c] | cyan[e] | m02145[c] | glcr[e] | m01681[c] |
| 3pg[e] | m00913[c] | cysi_L[e] | m01629[c] | glcur[e] | m01973[c] |
| 3ump[e] | m00921[c] | cys_L[e] | m01628[c] | glcur1p[e] | m01684[c] |
| 4abut[e] | m00970[c] | cytd[e] | m01630[c] | gln_L[e] | m01975[c] |
| 4hoxpac[e] | m01003[c] | dad_2[e] | m01666[c] | glu_L[e] | m01974[c] |
| 4hoxpacd[e] | m01002[c] | damp[e] | m01639[c] | gly[e] | m01986[c] |
| ac[e] | m01252[c] | dca[e] | m01648[c] | glyald[e] | m01981[c] |
| acac[e] | m01253[c] | dcmp[e] | m01644[c] | glyb[e] | m01393[c] |
| acald[e] | m01249[c] | dcyt[e] | m01668[c] | glyc[e] | m01983[c] |
| acgam[e] | m02527[c] | ddca[e] | m02344[c] | glyc3p[e] | m02914[c] |
| acgam1p[e] | m02528[c] | dgmp[e] | m01686[c] | glyclt[e] | m01998[c] |
| acmana[e] | m02524[c] | dgsn[e] | m01669[c] | glyc_R[e] | m01982[c] |
| acmum[e] | m02541[c] | dimp[e] | m01709[c] | gmp[e] | m02016[c] |
| acnam[e] | m02543[c] | din[e] | m01671[c] | gsn[e] | m02038[c] |
| acon_C[e] | m01580[c] | dopa[e] | m01736[c] | gthox[e] | m02027[c] |
| ade[e] | m01279[c] | drib[e] | m01672[c] | gthrd[e] | m02026[c] |
| adn[e] | m01280[c] | dtmp[e] | m01752[c] | gtp[e] | m02034[c] |
| AEP[e] | m00632[c] | dump[e] | m01755[c] | gua[e] | m02037[c] |
| agm[e] | m01303[c] | duri[e] | m01673[c] | h[e] | m02039[c] |
| akg[e] | m01306[c] | etha[e] | m01797[c] | h2o[e] | m02040[c] |
| alaala[e] | m03164[c] | etoh[e] | m01796[c] | h2o2[e] | m02041[c] |
| ala_B[e] | m01383[c] | f6p[e] | m01845[c] | h2s[e] | m02042[c] |
| ala_D[e] | m01638[c] | fald[e] | m01831[c] | hdca[e] | m02674[c] |
| ala_L[e] | m01307[c] | for[e] | m01833[c] | hdcea[e] | m02675[c] |
| alltn[e] | m01313[c] | fru[e] | m01840[c] | his_L[e] | m02125[c] |
| amp[e] | m01334[c] | fuc_L[e] | m01159[c] | hom_L[e] | m02136[c] |
| arab_L[e] | m02337[c] | fum[e] | m01862[c] | hxa[e] | m02120[c] |
| arg_L[e] | m01365[c] | g1p[e] | m01967[c] | hxan[e] | m02159[c] |
| ascb_L[e] | m01368[c] | g3pc[e] | m02912[c] | icit[e] | m02183[c] |
| asn_L[e] | m01369[c] | g3pe[e] | m02913[c] | ile_L[e] | m02184[c] |
| aso3[e] | m01367[c] | g3pi[e] | m02911[c] | imp[e] | m02167[c] |
| asp_L[e] | m01370[c] | g6p[e] | m01968[c] | inost[e] | m02171[c] |
| but[e] | m01410[c] | gal[e] | m01910[c] | ins[e] | m02170[c] |
| ca2[e] | m01413[c] | gal1p[e] | m01322[c] | k[e] | m02200[c] |
| cgly[e] | m01626[c] | galt[e] | m01909[c] | lac_D[e] | m01716[c] |
| chol[e] | m01513[c] | gam[e] | m01962[c] | lac_L[e] | m02403[c] |
| cit[e] | m01587[c] | gam6p[e] | m01963[c] | lcts[e] | m02332[c] |
| cmp[e] | m01590[c] | gbbtn[e] | m01922[c] | leu_L[e] | m02360[c] |
| co2[e] | m01596[c] | gdp[e] | m01948[c] | lys_L[e] | m02426[c] |
| mal_L[e] | m02439[c] | ser_L[e] | m02896[c] | dha[e] | m01984[s] |
| malt[e] | m02450[c] | so3[e] | m02949[c] | fe2[e] | m01821[c] |
| malthx[e] | m02447[c] | so4[e] | m02946[c] | fe3[e] | m01822[c] |
| malttr[e] | m02452[c] | spmd[e] | m02923[c] | maltpt[e] | m02449[c] |
| maltttr[e] | m02451[c] | succ[e] | m02943[c] | ocdcea[e] | m02646[c] |
| man[e] | m02453[c] | tag_D[e] | m01745[c] | orn[e] | m02658[c] |
| man6p[e] | m02455[c] | taur[e] | m02961[c] | tre[e] | m03039[s] |
| met_L[e] | m02471[c] | tcynt[e] | m02986[c] | ttdcea[e] | m00117[c] |
| mg2[e] | m02482[c] | thm[e] | m02982[c] | guln_L[e] | m02378[c] |
| minohp[e] | m02492[c] | thr_L[e] | m02993[c] | cu2[e] | m01624[c] |
| na1[e] | m02519[c] | thym[e] | m02997[c] |  |  |
| nac[e] | m02586[c] | thymd[e] | m02996[c] |  |  |
| nh4[e] | m02579[c] | tma[e] | m03053[c] |  |  |
| nmn[e] | m02581[c] | tmao[e] | m03054[c] |  |  |
| no[e] | m02609[c] | trp_L[e] | m03089[c] |  |  |
| no2[e] | m02588[c] | tsul[e] | m02991[c] |  |  |
| o2[e] | m02630[c] | ttdca[e] | m02494[c] |  |  |
| o2s[e] | m02631[c] | tym[e] | m03099[c] |  |  |
| oaa[e] | m02633[c] | tyr_L[e] | m03101[c] |  |  |
| ocdca[e] | m02938[c] | uacgam[e] | m03111[c] |  |  |
| octa[e] | m02642[c] | udpacgal[e] | m03110[c] |  |  |
| orot[e] | m02659[c] | udpg[e] | m03108[c] |  |  |
| pacald[e] | m02719[c] | udpgal[e] | m03107[c] |  |  |
| peamn[e] | m02718[c] | udpglcur[e] | m03109[c] |  |  |
| pep[e] | m02696[c] | ump[e] | m03114[c] |  |  |
| phe_L[e] | m02724[c] | ura[e] | m03118[c] |  |  |
| pheme[e] | m02049[c] | urea[e] | m03121[c] |  |  |
| pi[e] | m02751[c] | uri[e] | m03123[c] |  |  |
| pnto_R[e] | m02680[c] | val_L[e] | m03135[c] |  |  |
| ppa[e] | m02772[c] | xan[e] | m03148[c] |  |  |
| pro_L[e] | m02770[c] | xmp[e] | m03150[c] |  |  |
| pser_L[e] | m00916[c] | xtsn[e] | m03149[c] |  |  |
| ptrc[e] | m02812[c] | xyl_D[e] | m01758[c] |  |  |
| pydx[e] | m02813[c] | xylu_L[e] | m02425[c] |  |  |
| pydxn[e] | m02817[c] | zn2[e] | m03157[c] |  |  |
| pyr[e] | m02819[c] | chitob[e] | m01439[l] |  |  |
| r5p[e] | m02845[c] | 12ppd_R[e] | m02771[c] |  |  |
| rib_D[e] | m02843[c] | acgal1p[e] | m02526[c] |  |  |
| rnam[e] | m02582[c] | acgal[e] | m02525[c] |  |  |
| sbt_D[e] | m01682[c] | adocbl[e] | m01600[m] |  |  |
| ser_D[e] | m01744[c] | cl[e] | m01442[c] |  |  |
