# Supplementary material for: Dual transcriptome based reconstruction of Salmonella-human integrated metabolic network to screen potential drug targets
Source: PLoS One. 2022 May 24;17(5):e0268889. doi: 10.1371/journal.pone.0268889 (PMC9129043; doi:10.1371/journal.pone.0268889)
Supplement: S8 Table — (DOCX) [file pone.0268889.s017.docx]

S8 Table. DMEM medium constraints for the host GMN used in metabolic model simulations

| Reactionname | Lower bound | Upper bound | Mets | RXN_Formula |
| --- | --- | --- | --- | --- |
| H2O exchange | -1000 | 1000 | m02040[s] | m02040[s] <=> |
| O2 exchange | -1000 | 1000 | m02630[s] | m02630[s] <=> |
| arginine exchange | -1000 | 1000 | m01365[s] | m01365[s] <=> |
| chloride exchange | -1000 | 1000 | m01442[s] | m01442[s] <=> |
| pantothenate exchange | -1000 | 1000 | m02680[s] | m02680[s] <=> |
| folate exchange | -1000 | 1000 | m01830[s] | m01830[s] <=> |
| tyrosine exchange | -1000 | 1000 | m03101[s] | m03101[s] <=> |
| linolenate exchange | -1000 | 1000 | m02389[s] | m02389[s] <=> |
| serine exchange | -1000 | 1000 | m02896[s] | m02896[s] <=> |
| cystine exchange | -1000 | 1000 | m01629[s] | m01629[s] -> |
| glycine exchange | -1000 | 1000 | m01986[s] | m01986[s] <=> |
| ascorbate exchange | -1000 | 1000 | m01368[s] | m01368[s] <=> |
| pyridoxine exchange | -1000 | 1000 | m02817[s] | m02817[s] -> |
| thiamin exchange | -1000 | 1000 | m02982[s] | m02982[s] <=> |
| linoleate exchange | -1000 | 1000 | m02387[s] | m02387[s] <=> |
| phenylalanine exchange | -1000 | 1000 | m02724[s] | m02724[s] <=> |
| valine exchange | -1000 | 1000 | m03135[s] | m03135[s] <=> |
| riboflavin exchange | -1000 | 1000 | m02842[s] | m02842[s] <=> |
| glucose exchange | -1000 | 1000 | m01965[s] | m01965[s] <=> |
| threonine exchange | -1000 | 1000 | m02993[s] | m02993[s] <=> |
| tryptophan exchange | -1000 | 1000 | m03089[s] | m03089[s] <=> |
| glutamine exchange | -1000 | 1000 | m01975[s] | m01975[s] <=> |
| Na+ exchange | -1000 | 1000 | m02519[s] | m02519[s] <=> |
| K+ exchange | -1000 | 1000 | m02200[s] | m02200[s] <=> |
| nicotinate exchange | -1000 | 1000 | m02586[s] | m02586[s] <=> |
| histidine exchange | -1000 | 1000 | m02125[s] | m02125[s] <=> |
| isoleucine exchange | -1000 | 1000 | m02184[s] | m02184[s] <=> |
| leucine exchange | -1000 | 1000 | m02360[s] | m02360[s] <=> |
| lysine exchange | -1000 | 1000 | m02426[s] | m02426[s] <=> |
| methionine exchange | -1000 | 1000 | m02471[s] | m02471[s] <=> |
| Pi exchange | -1000 | 1000 | m02751[s] | m02751[s] <=> |
| retinol exchange | -1000 | 1000 | m02834[s] | m02834[s] <=> |
