# Supplementary material for: Dual transcriptome based reconstruction of Salmonella-human integrated metabolic network to screen potential drug targets
Source: PLoS One. 2022 May 24;17(5):e0268889. doi: 10.1371/journal.pone.0268889 (PMC9129043; doi:10.1371/journal.pone.0268889)
Supplement: S9 Table — (DOCX) [file pone.0268889.s018.docx]

S9 Table. Long names of metabolites given in S1 Figure, the figüre that reports metabolite-metabolite interactions around pabB catalyzed reaction.

| **Metabolite Name** | **Abbrevation** | **Metabolite Name** | **Abbrevation** |
| --- | --- | --- | --- |
| O-Phospho-4-hydroxy-L-threonine | phthr[c] | 2-3-dihydroxybenzoylserine | 23dhbzs[c] |
| D-Ribulose-5-phosphate | ru5p_D[c] | L-Serine | ser_L[c] |
| 4-Aminobutanoate | 4abut[c] | 7-8-Dihydrofolate | dhf[c] |
| 2-Oxoglutarate | akg[c] | Dihydropteroate | dhpt[c] |
| L-Glutamate | glu_L[c] | 6-hydroxymethyl-dihydropterin-pyrophosphate | 6hmhptpp[c] |
| Coenzyme-A | coa[c] | 2-3-diketo-5-methylthio-1-phosphopentane | dkmpp[c] |
| GTP | gtp[c] | Glyceraldehyde-3-phosphate | g3p[c] |
| Bicarbonate | hco3[c] | 2-Aminomalonate-semialdehyde | 2amsa[c] |
| Pyruvate | pyr[c] | dTMP | dtmp[c] |
| N-Acetyl-L-glutamate | acglu[c] | dUMP | dump[c] |
| 2-Oxobutanoate | 2obut[c] | 2-Deoxy-D-ribose-1-phosphate | 2dr1p[c] |
| N2-Acetyl-L-ornithine | acorn[c] | L-seryl-AMP | seramp[c] |
| O-Acetyl-L-serine | acser[c] | 5-10-Methylenetetrahydrofolate | mlthf[c] |
| Sodium | na1[p] | L-Phenylalanine | phe_L[c] |
| 4-Aminobenzoate | 4abz[c] | L-Leucine | leu_L[c] |
| L-Glutamine | gln_L[c] | L-Tyrosine | tyr_L[c] |
| chorismate | chor[c] | L-Isoleucine | ile_L[c] |
| 5-Phospho-alpha-D-ribose-1-diphosphate | prpp[c] | L-Valine | val_L[c] |
| L-Aspartate | asp_L[c] | L-Tryptophan | trp_L[c] |
| Putrescine | ptrc[c] | D-Fructose-6-phosphate | f6p[c] |
| 10-Formyltetrahydrofolate | 10fthf[c] | Formate | for[p] |
| 5-6-7-8-Tetrahydrofolate | thf[c] | L-Methionyl-tRNA-Met | mettrna[c] |
| 5-amino-1-5-phospho-D-ribosyl-imidazole | air[c] | Glycerophosphoserine | g3ps[c] |
| L-alanine-L-glutamate | LalaLglu[c] | 1-Pyrroline-5-carboxylate | 1pyr5c[c] |
| L-Alanine | ala_L[c] | L-Glutamate-5-phosphate | glu5p[c] |
| Formate | for[c] | N1-5-Phospho-D-ribosyl-glycinamide | gar[c] |
| Glycine | gly[c] | N2-Formyl-N1-5-phospho-D-ribosyl-glycinamide | fgam[c] |
| Anthranilate | anth[c] | gamma-L-Glutamyl-L-cysteine | glucys[c] |
| L-Methionine | met_L[c] | L-Glutamate | glu_L[p] |
| L-Homocysteine | hcys_L[c] | D-Glutamate | glu_D[c] |
| 3-Methyl-2-oxobutanoate | 3mob[c] | L-Glutamyl-tRNA-Glu | glutrna[c] |
| Adenosyl-cobyrinate-a-c-diamide | adcob1nda[c] | N-Formimino-L-glutamate | nflgln[c] |
| 4adcho[c] | 4-amino-4-deoxychorismate | O-Phospho-L-serine | pser_L[c] |
| Xanthosine-5-phosphate | xmp[c] | N2-Succinyl-L-ornithine | sucorn[c] |
| L-Serine | ser_L[p] | N-Succinyl-LL-2-6-diaminoheptanedioate | sl26da[c] |
| L-Histidinol-phosphate | hisp[c] | L-Seryl-tRNA-Sec | sertrna_sec_[c] |
| 5-5-phospho-1-deoxyribulos-1-ylamino-methylideneamino-1-5-phosphoribosyl-imidazole-4-carboxamide | prlp[c] | L-Seryl-tRNA-Ser | sertrna[c] |
| 5-Methyltetrahydrofolate | 5mthf[c] | S-Formylglutathione | Sfglutth[c] |
| 2-Dehydropantoate | 2dhp[c] | N2-Succinyl-L-glutamate | sucglu[c] |
| 5-10-Methenyltetrahydrofolate | methf[c] | dTDP-4-amino-4-6-dideoxy-D-galactose | dtdp4addg[c] |
| Thymidine | thymd[c] | Thymine | thym[c] |
| Thymidine | thymd[p] | Thymine | thym[p] |
| phosphatidylserine-didodecanoyl-n-C120 | ps120[c] | uridine-5--diphospho-beta-4-deoxy-4-amino-L-arabinose | udpLa4n[c] |
| phosphatidylserine-ditetradecanoyl-n-C140 | ps140[c] | undecaprenyl-phosphate-4-amino-4-formyl-L-arabinose | uLa4fn[c] |
| phosphatidylserine-ditetradec-7-enoyl-n-C141 | ps141[c] | Cobyrinate | cobn[c] |
| phosphatidylserine-dihexadecanoyl-n-C160 | ps160[c] | 4-Aminobenzoate | 4abz[e] |
| phosphatidylserine-dihexadec-9-enoyl-n-C161 | ps161[c] | R-Pantothenate | pnto_R[c] |
| phosphatidylserine-dioctadecanoyl-n-C180 | ps180[c] | R-Pantoate | pant_R[c] |
| phosphatidylserine-dioctadec-11-enoyl-n-C181 | ps181[c] |  |  |
