# Supplementary material for: Dual transcriptome based reconstruction of Salmonella-human integrated metabolic network to screen potential drug targets
Source: PLoS One. 2022 May 24;17(5):e0268889. doi: 10.1371/journal.pone.0268889 (PMC9129043; doi:10.1371/journal.pone.0268889)
Supplement: Attachment — Submitted filename: Response to reviewers.pdf [file pone.0268889.s021.pdf]

Replies for Reviewer Comments

Reviewer 2:

1) The authors have addressed the comments. However, it will be worthwhile to show the data mentioned in Comment -9. It is important to see the early changes (8h time point) in the metabolic flux irrespective of how small they are.

We thank the reviewer for this comment. The number of reaction and metabolite information of the reconstructed GMN model for the early infection (8h time point) were added to Table 1 in the Results section. The predicted by-product secretion rates based on this metabolic model were also added to Table 2. We also updated Supplementary Figure 8 (PCA of dual transcriptome data) by including the samples of 8th hour of infection.
